# Maternal, childhood and adolescent influences on Leydig cell functional capacity and circulating INSL3 concentration in young adults: Importance of childhood infections and body mass index

**DOI:** 10.1111/andr.70091

**Published:** 2025-07-02

**Authors:** Richard Ivell, Bilal Tulumcu, Waleed Alhujaili, Ravinder Anand‐Ivell

**Affiliations:** ^1^ School of Biosciences University of Nottingham Sutton Bonington Loughborough UK; ^2^ Present address: Department of Biology College of Science Taibah University Medina Saudi Arabia

**Keywords:** ALSPAC, chickenpox, hypogonadism, inflammation, INSL3, Leydig cell, obesity

## Abstract

**Background:**

The constitutive Leydig cell hormone insulin‐like peptide 3 (INSL3) is considered a good estimate of the adult Leydig cell functional capacity and appears to remain relatively consistent throughout adult male life, only gradually declining into old age. Importantly, in younger men it appears to predict hypogonadism and hence later health and morbidity.

**Objectives:**

Here, we have used the Avon Longitudinal Study of Parents and Children (ALSPAC) cohort of boys and young men to assess those factors during pregnancy, infancy, childhood, and adolescence, when the adult‐type Leydig cell population is being established, which by association might influence the final circulating INSL3 concentration in young adulthood.

**Methods:**

A wide range of clinical, anthropometric and lifestyle parameters were assessed from up to 2000 boys and young men based on direct medical measurement and/or by targeted questionnaires. Scalar variables used bivariate correlation analysis, whereas comparative statistics, such as *t*‐tests, were applied to categorical variables.

**Results:**

Maternal parameters, such as maternal smoking, gestational age, or being small for gestational age (SGA) appeared to have no association with adult INSL3 levels. Of all the parameters assessed, those with greatest impact on young adult Leydig cell status appeared to be childhood and adolescent body mass index (BMI) and early childhood infectious disease. Particularly, chickenpox in infancy had a marked and significant negative association with INSL3 (reduced by 10%–14%) at both 17 and 24 years. Being overweight (> 85th percentile) at 13 years was associated with a 20% reduction in young adult INSL3. In contrast, childhood and adolescent inflammatory factors and cigarette exposure appeared to have no long‐lasting impact on adult Leydig cell status.

**Limitations:**

This retrospective cohort study is limited by relatively small numbers and by its correlative analysis. The hypotheses generated will need to be validated in more extensive, in‐depth studies.

**Lay summary:**

The testis hormone INSL3 is considered a good estimate of the gonadal capacity to make testosterone. It is relatively constant throughout adult male life, only gradually declining into old age. Importantly, in younger men reduced INSL3 appears to predict hypogonadism and hence later health and morbidity. Whilst showing low within‐individual variation, between individuals INSL3 can vary more than 10‐ up to 100‐fold. The source of this variance is unknown but is believed to have its origin during childhood and adolescence. Analysis of data from the ALSPAC cohort implies that of various parameters assessed, only childhood and adolescent BMI as well as early infectious disease, in particular chickenpox, have any influence on young adult INSL3 concentration, respectively reducing circulating levels each by 10%–20%. More research is needed to understand the mechanisms how these factors influence testis function, what can be done as mitigation, and what other factors may be involved.

## 1 INTRODUCTION

In men, the testicular Leydig cells are responsible for producing > 95% of the testosterone required for maintaining male‐specific physiology during adult life. These cells develop during puberty from mesenchymal stem cells in the testes, beginning at around 8–9 years of age.^1^, ^2^ They proliferate and differentiate under the influence of pituitary Luteinising Hormone (LH), first to form so‐called progenitor, then immature Leydig cells, and finally terminally differentiated adult‐type Leydig cells at the end of puberty. The population of Leydig cells at the end of puberty appears then no longer to divide and remains stable for the remainder of the male lifespan, with little or no attrition. Within a population, the Leydig cell functional capacity to make androgens like testosterone varies greatly between individuals and can be assessed by measuring the concentration of the Leydig cell hormone Insulin‐like peptide 3 (INSL3) in the circulation. INSL3 is secreted by mature Leydig cells in a constitutive manner and shows very little variance within individuals over periods of months or years.^3^, ^4^ In middle‐aged and older men circulating INSL3 predicts age‐related morbidity, such as diabetes, cardiovascular disease, or low bone density, and offers a good index of hypogonadism.^5^ Longitudinal studies suggest that the broad (> 10‐fold) variance in INSL3 concentration between individuals is already established in young adulthood,^6^, ^7^ and that whatever factors are responsible for establishing this variance are therefore potentially at least in part causative for morbidity in older age.

The Avon Longitudinal Study of Parents And Children (ALSPAC) has assembled a broad range of maternal, childhood, and adolescent clinical and lifestyle parameters for a cohort of children born in the early nineties and now in young adulthood.^8^, ^9^ Blood samples were measured for INSL3 from approximately 2000 young men from the ALSPAC cohort aged at about 17 and/or 24 years.^10^ We were able to show that at 17 years pubertal development of the Leydig cell population was still ongoing with maximal INSL3 values mostly not yet attained. In contrast, at 24 years, the Leydig cell population had dynamically stabilised to represent the adult Leydig cell population likely to pertain for the remainder of the lifespan.^10^ Cross‐sectional correlation analysis suggested that of the contemporary parameters assessed at 24 years only body mass index (BMI) and smoking incidence were influential to a modest extent, although the INSL3 concentration at 17 years for the same individuals also contributed to the INSL3 variance at 24 years. In this preceding study,^10^ we also showed that INSL3 in young adulthood was not dependent on the timing of puberty measured using the parameter age at peak height velocity, nor did the latter affect the timing of peak INSL3. Taken together, the results implied that mostly factors prevalent during or before puberty were instrumental in determining Leydig cell functional capacity in young adult men, and hence their future risk of later age‐related morbidity.

Here, we have extended the earlier study^10^ to report the relationships between INSL3 concentration in young men at 17 and 24 years and a range of maternal, childhood, and adolescent parameters for the same individuals, and show that of those assessed, childhood BMI and common childhood infections appeared to be of greatest long‐term consequence.

## 2 METHODS AND MATERIALS

The ALSPAC is a forward‐looking, population‐based birth cohort study carried out in the Bristol area of the United Kingdom. Designed to examine the factors influencing health and development throughout the lifespan, ALSPAC enrolled pregnant women living in the former county of Avon who were expected to give birth between April 1991 and December 1992.^11^, ^12^ The initial cohort included 14,541 pregnancies. This was supplemented later by further children recruited at around age 7 years, so that the total sample size for data analysis post 7 years reflected a total of 15,447 pregnancies. Of these, 14,901 children were alive at 1 year of age. A wide range of data has been collected from participants via questionnaires, clinical assessments, biological samples, and linkage to medical records (for more details, see www.bristol.ac.uk/alspac/researchers/our‐data/). In this study, we utilised data from 1,781 young men in the ALSPAC cohort whose blood samples at ages 17 and 24 years were available for INSL3 analysis (see below). For the 24 year subjects, data were collected and managed using REDCap electronic data capture tools hosted at the University of Bristol.^13^, ^14^ REDCap (Research Electronic Data Capture) is a secure, web‐based software platform designed to support data capture for research studies. Only data for subjects indicating removal of one or both testes or having severe congenital disease (e.g., trisomy) were excluded. Blood analyses, anthropometric measurements, clinical assessments and questionnaire data had been collected from these participants at various ages, including some maternal parameters.

### 2.1 Maternal and pregnancy‐related parameters

Birthweight was collected as part of the obstetric data, which also included placental weight. Gestational age was calculated (in days) based on the date of the last menstrual period (LMP) with ultrasound assessment for uncertain LMP dates and/or discrepancies with clinical evaluation. Births were considered small for gestational age (SGA) when birthweights were less than the 10th percentile for this cohort. Maternal age was calculated from birth date to age at delivery. The ratio of the lengths of the second to fourth digits of left and right hands (2D:4D ratio) is considered to reflect any androgen–estrogen imbalance during pregnancy.^15^ It was assessed at age 11 years from photocopies, with finger length measured using a “Mahr Digital Caliper 16 EX” with 0.01 mm precision. Maternal smoking data were obtained from questionnaires administered to mothers during the 18th–20th weeks of pregnancy.

### 2.2 Infancy‐related parameters

Neonatal weight gain data were obtained at three different time intervals for the infants (from birth to 6–8 weeks, from 6–8 weeks to 9 months, and from birth to 9 months and 18 months). Using the UK 1990 Growth Reference, infant weights were converted into *z*‐scores for each interval incorporating Cole's procedure.^16^ Positive values indicate faster weight gain than average, whereas negative values indicate slower weight gain than average. Breastfeeding data were obtained from a questionnaire administered to the mother when the infant was 6 months old. A composite weaning index (CFUI; Complementary Feeding Utility Index) was also applied comprising 14 components including breastfeeding duration, timing of introduction to solids, and quality of solid food intake based on questionnaires at 3 and 6 months.^17^

### 2.3 Childhood and adolescence anthropometric and endocrine parameters

BMI and height data were obtained from questionnaires sent to mothers each year when participants were between 8 and 16 years old; at 17 years, these were measured during clinical assessment using a Harpenden stadiometer and a Tanita body fat analyzer (model TBF 401A).

Peripheral venous blood samples were collected during these clinical visits with serum or plasma aliquoted and stored frozen at −80C until analyses. INSL3 was measured using a highly specific time‐resolved fluorescent immunoassay (TRFIA), as described,^3^ at 17 and 24 years. Cotinine was assayed in plasma using the Cozart Cotinine Enzyme Immunoassay (Concateno (now Alere Toxicology), Abingdon, UK) serum kit (M155B1). thyroid stimulating hormone (TSH), free T3, and free T4 were measured using a Cobas e601 system (Roche Diagnostics, Mannheim, Germany). Interleukin 6 (IL‐6), leptin and C‐reactive protein (CRP) were analysed in different departments at the Glasgow Royal Infirmary.^18^ CRP was assessed by automated particle‐enhanced immunoturbidimetric assay (Roche Diagnostics, Burgess Hill, UK). Steroid hormone binding globulin (SHBG) and leptin were measured by ELISA (DRG Instruments GmbH, Marburg, Germany), cortisol by chemiluminescent ELISA (DSL; Oxford, UK), whereas IL‐8 and transforming growth factor alpha (TNFα) made use of Olink Proteomics Proximity Extension Assay (PEA) technology (Olink Proteomics, Uppsala, Sweden).

### 2.4 Childhood infectious diseases

All childhood infection data were obtained via questionnaires sent to the children's caregivers beginning when the children were 18 months old and continued at specified intervals until children were 13 years old (157th month).

### 2.5 Statistical analysis

All statistical analyses were performed using R software (version 2024.05.0), GraphPad Prism (version 10.3.1), or SPSS (version 29.02.0). Variables exhibiting significant skewness were log‐transformed prior to analysis to approximate normality. Simple bivariate correlation analyses were conducted to evaluate relationships between INSL3 concentrations at ages 17 and 24 years and maternal/neonatal factors, with multiple regression analysis applied to non‐collinear parameters indicating significant correlation with INSL3 at *p* < 0.1 (inclusion threshold). Since this was an exploratory study, type 1 errors due to multiple comparisons were not taken into account in the presented results; however, Benjamini–Hochberg adjustment was subsequently applied to monitor such errors and except for the maternal factors (Table 1) all significant parameters remained significant, as shown. Following ALSPAC guidelines, cell values ≤ 5 were reported as such to preclude any possibility of subject identification. *t*‐Tests were performed to compare mean INSL3 levels across categorical groups (e.g., maternal smoking or childhood illnesses), with Welch's correction applied where variance was non‐homogeneous. All statistical tests were two‐sided, and a *p*‐value < 0.05 was considered statistically relevant.

**TABLE 1:** Maternal and neonatal factors.

A. Bivariate correlation analysis						
	INSL3 at 17 years			INSL3 at 24 years		
	Pearson correlation	Significance (*p*‐value)	*N*	Pearson correlation	Significance (*p*‐value)	*N*
Birthweight	−0.059	**0.037**	1232	0.023	0.473	1165
Gestational age	−0.061	**0.031**	1247	−0.001	0.991	1176
Placental weight	−0.056	0.341	289	0.086	0.161	266
Maternal age	0.036	0.203	1247	−0.005	0.860	1176
Weaning index	−0.047	0.129	1042	−0.005	0.882	994
Left 2D/4D ratio	0.028	0.344	1113	−0.005	0.885	1032
Right 2D/4D ratio	0.023	0.452	1113	−0.009	0.780	1032
Weight gain (0–6w)	−0.018	0.645	653	−0.052	0.194	626
Weight gain (0–9 m)	0.027	0.481	668	−0.026	0.514	638
Weight gain (0–18 m)	0.038	0.350	616	0.028	0.491	590
Weight gain (6w–9 m)	0.013	0.751	625	−0.026	0.517	602
Weight gain (6w–18 m)	0.037	0.373	581	0.010	0.810	560
Weight gain (9–18 m)	0.005	0.903	580	−0.073	0.083	559

B. Categorical comparisons						
	INSL3 at 17 years			INSL3 at 24 years		
	Mean ± SD	*N*	*p*‐value	Mean ± SD	*N*	*p*‐value
Maternal smoking before pregnancy						
Yes	1.54 ± 2.45	273	0.646	2.21 ± 1.76	255	0.658
Never	1.47 ± 1.57	954		2.15 ± 1.49	905	
Maternal smoking in first trimester						
Yes	1.34 ± 1.31	187	0.125	2.14 ± 1.64	178	0.829
Never	1.51 ± 1.88	1040		2.17 ± 1.54	983	
SGA (small for gestational age)						
<10th %ile	1.72 ± 2.95	117	0.331	2.13 ± 1.21	97	0.766
≥10th %ile	1.45 ± 1.63	1133		2.17 ± 1.58	1080	
Breastfeeding						
Yes	1.43 ± 1.82	394	0.242	2.08 ± 1.41	402	0.090
Never	1.75 ± 3.05	145		2.43 ± 2.14	127	

*Note*: (A) Bivariate correlation analysis. (B) Categorical comparisons. Results highlighted in red are significant at *p* < 0.05, though Benjamini–Hochberg adjustment for multiple comparisons eliminated this significance.

### 2.6 Ethical considerations

The study was approved by the ALSPAC Ethics and Law Committee, and written informed consent for biological samples was obtained from all participants, in accordance with the UK Human Tissue Act 2004. Informed consent for the use of data collected via questionnaires and clinics was obtained from participants following the recommendations of the ALSPAC Ethics and Law Committee at the time. All data were fully anonymised. Detailed descriptions of the cohort and study design have been previously published and are accessible on the ALSPAC website (www.bristol.ac.uk/alspac/researchers/our‐data/) which also provides comprehensive details of all available parameters through a fully searchable data dictionary and variable search tool.

## 3 RESULTS

### 3.1 Influence of maternal, pregnancy and infancy‐related parameters

Both birthweight and gestational age were negatively correlated with the INSL3 concentration at 17 years of age, but not at 24 years of age (Table 1A), though the correlation coefficients are small. Moreover, these correlations lost significance when adjusted for multiple comparisons (not shown). Maternal age at delivery showed no relationship with INSL3 concentration at either age. When births were categorised as being SGA (< 10th percentile) or not, then a *t*‐test indicated no impact on INSL3 concentration at either 17 years or 24 years (Table 1B).

Questionnaires were used to determine whether or not the mothers smoked before pregnancy, or during the first trimester (Table 1B). There appeared to be no effect for either category on INSL3 at either 17 or 24 years. Placental weight was also available for a small number of births, though caution is warranted, since it is not recorded whether or not this included the weight of the umbilical cord. Correlation analysis indicated no impact of registered placental weight on INSL3 concentration at 17 or 24 years.

The ALSPAC dataset also includes measurements of the 2nd (2D) and 4th (4D) digits of the right and left hands taken during childhood. The 2D/4D ratio differs between male and female sex, and in boys has been considered as a possible indication for an altered testosterone‐estrogen balance during pregnancy, for example, possibly caused by maternal exposure to environmental endocrine disrupting substances.^15^ Bivariate correlation analysis (Table 1A) showed no relationship of the 2D/4D ratio (left or right hand) to INSL3 concentration at either 17 or 24 years.

Finally, questionnaire data were available for both breastfeeding (Table 1B) and a weaning index (Table 1A). A *t*‐test comparison between babies exclusively breastfed and those never breastfed during the first 2 months of life showed no difference with regard to INSL3 concentration at either 17 years or 24 years. Similarly, there was no association for INSL3 at either age with the so‐called weaning index (Table 1A). An apparent lack of nutritional impact in infancy on later INSL3 concentration was mostly also supported by the bivariate correlation analysis between INSL3 at 17 and 24 years and an index of infant weight gain from birth to 6 weeks, 9 months, and 18 months (Table 1A), although high weight gain between 9 and 18 months suggested a tendency (*p* < 0.083) for negative impact on the INSL3 concentration at 24 years (Table 1A).

### 3.2 Influence of childhood and adolescent anthropometric and lifestyle parameters

Table 2 shows the results of bivariate correlation analysis between circulating INSL3 concentration at 17 and 24 years and simple anthropometric parameters of height and BMI (weight alone showed no relationship). Importantly, INSL3 concentration at 17 years correlated positively with height at 13 years of age and also negatively with BMI at both 10 and 13 years of age (Table 2A and 2B). Although INSL3 at 24 years of age indicated no relationship to height alone, it did show a negative association (*p* < 0.05) with BMI at 9, 13 and 17 years and a tendency towards association (*p* < 0.1) at 11, 14 and 16 years (Table 2B). It is to be noted that there was no further significant association for INSL3 at 17 years with either height or BMI at any other age prior to 17 years during childhood and adolescence. However, BMI at all ages correlates with one another as well as with calculated calory intake at 3, 7 and 13 years, though not with neonatal weight gain (Table S1), so that generally it appears that reduced INSL3 concentration at both 17 and 24 years is strongly and negatively associated with being overweight through childhood and adolescence. In fact, when BMI at 13 years is taken as indicator and segregated into those individuals who could be classified as clinically overweight (> 85th percentile; BMI > 22.4) and a control group comprising subjects < 50th percentile, then a *t*‐test shows that being overweight at this age markedly impacts later INSL3 concentration at both 17 and 24 years (Table 2C) and leads to a 20% reduction in adult INSL3 concentration.

**TABLE 2:** Bivariate correlation analysis in childhood and adolescence.

A. Height						
	INSL3 at 17 years			INSL3 at 24 years		
	Pearson correlation	Significance (*p*‐value)	*N*	Pearson correlation	Significance (*p*‐value)	*N*
Height 8y	0.008	0.830	685	−0.026	0.516	644
Height 9y	0.059	0.104	751	−0.005	0.899	702
Height 10y	0.030	0.427	699	−0.031	0.424	684
Height 11y	0.061	0.096	741	0.010	0.788	708
Height 13y	0.116	**0.002**	730	0.041	0.270	714
Height 14y	0.040	0.277	742	0.000	0.992	723
Height 16y	0.038	0.320	705	0.043	0.266	668
Height 17y	0.030	0.439	687	0.026	0.498	663

B. BMI						
	INSL3 at 17 years			INSL3 at 24 years		
	Pearson correlation	Significance (*p*‐value)	*N*	Pearson correlation	Significance (*p*‐value)	*N*
BMI 8y	0.039	0.335	605	0.000	0.996	579
BMI 9y	−0.012	0.765	669	−0.111	**0.005**	632
BMI 10y	−0.109	**0.005**	651	−0.038	0.338	632
BMI 11y	0.019	0.623	664	−0.066	0.094	646
BMI 13y	−0.111	**0.004**	665	−0.101	**0.010**	662
BMI 14y	−0.052	0.174	687	−0.073	0.059	675
BMI 16y	−0.045	0.248	668	−0.070	0.079	633
BMI 17y	−0.041	0.299	658	−0.092	**0.019**	643

C. Categorical comparison of being overweight at 13 years.						
	INSL3 at 17 years			INSL3 at 24 years		
	Mean ± SD	*N*	*p*‐value	Mean ± SD	*N*	*p*‐value
Overweight (> 85th percentile) based on BMI at 13 years	1.23 ± 1.09	97	**<0.01**	1.82 ± 1.04	98	**<0.001**
Control (< 50th percentile) based on BMI at 13 years	1.60 ± 1.78	297		2.26 ± 1.48	341	

D. Cotinine						
	INSL3 at 17 years			INSL3 at 24 years		
	Pearson correlation	Significance (*p*‐value)	*N*	Pearson correlation	Significance (*p*‐value)	*N*
Cotinine 7y	0.012	0.743	709	0.003	0.942	650
Cotinine 15y	−0.019	0.633	633	−0.073	0.092	538

E. Endocrine parameters						
	INSL3 at 17 years			INSL3 at 24 years		
	Pearson correlation	Significance (*p*‐value)	*N*	Pearson correlation	Significance (*p*‐value)	*N*
Free T3 (7y)	−0.007	0.856	718	−0.003	0.945	647
Free T3 (11y)	−0.024	0.789	129	−0.076	0.415	118
Free T3 (15y)	0.042	0.395	406	0.014	0.789	396
Free T4 (7y)	0.004	0.916	723	−0.018	0.645	652
Free T4 (11y)	0.106	0.228	132	−0.024	0.794	120
Free T4 (15y)	0.117	**0.019**	408	−0.018	0.726	397
TSH (7y)	−0.045	0.227	722	0.014	0.731	649
TSH (11y)	0.042	0.636	131	−0.0913	0.882	120
TSH (15y)	0.043	0.385	416	0.021	0.671	400
IL6 (9y)	0.043	0.205	867	−0.051	0.149	772
IL8 (9y)	0.029	0.455	686	0.065	0.078	726
CRP (9y)	0.049	0.146	870	−0.045	0.210	774
CRP (15y)	−0.044	0.196	862	−0.002	0.964	729
Leptin (9y)	0.134	0.059	198	−0.002	0.983	167
SHBG (9y)	−0.073	0.310	198	0.101	0.194	186
TNFα (9y)	0.022	0.555	686	0.061	0.097	726
Cortisol (8.5y)	0.167	**0.018**	201	0.039	0.609	171
Cortisol (15y)	0.096	**0.033**	494	0.037	0.446	418

F. Effect of early or late‐onset inflammation						
	INSL3 at 17 years			INSL3 at 24 years		
	Mean ± SD	*N*	*p*‐value	Mean ± SD	*N*	*p*‐value
Early inflammation						
(9 vs. 15 and/or 17 years)	1.63 ± 1.47	111	0.215	2.09 ± 1.19	85	0.129
Late inflammation						
(9 vs. 15 and/or 17 years)	1.45 ± 1.49	689		2.32 ± 1.80	513	

*Note*: (A) INSL3 at 17 and 24 years vs. height at annual intervals. (B) INSL3 at 17 and 24 years vs. BMI at annual intervals. (C) Categorical comparison of INSL3 at 17 and 24 years with being overweight (> 85th percentile) at 13 years vs. control (< 50th percentile at 13 years). (D) Correlation between INSL3 at 17 and 24 years and serum cotinine concentration at 7 and 15 years. (E) Correlation between INSL3 at 17 and 24 years and endocrine parameters at various ages as indicated (T3, triiodothyronine; T4, thyroxine; TSH, thyroid stimulating hormone; IL6, interleukin 6; IL8, interleukin 8; CRP, C‐reactive protein; SHBG, steroid hormone binding globulin; TNFα, transforming growth factor alpha). (F) Effect of early or late‐onset inflammation following the criteria of Palmer et al.,^19^ compared using *t*‐test. Results highlighted in red are significant at *p* < 0.05; these all retained significance after adjustment for multiple comparisons.

Indirect evidence of smoking exposure was available via blood cotinine levels measured at 7 and 15 years of age (Table 2D). However, this parameter at either age appears to have no influence on the INSL3 concentration at 17 and 24 years.

Some endocrine parameters were also available for the boys in the ALSPAC cohort during childhood and adolescence (Table 2E). Of these, only free T4 (thyroxine) at 15 years, and cortisol at age 8.5 years and also at 15 years, indicated any association, and then only with INSL3 at 17 years. There were no associations for any other available endocrine parameters with INSL3 at 17 or 24 years, including a range of inflammation markers (Table 2E), though IL8 and TNFα at 9 years suggested a tendency (< 0.10) towards a positive relationship (Table 2E). Previously, CRP (C‐reactive protein) from the ALSPAC dataset was used to classify subjects as having either early or late‐onset inflammation in the context of lasting psychological or cardiovascular disturbance.^19^ Similar categorisation was also used here to determine any possible association of inflammation with later INSL3 concentration (Table 2F). No effect was evident.

### 3.3 Influence of childhood infectious diseases

It is known that contemporary viral infection for HIV or COVID19 can affect Leydig cell function leading to reduced circulating testosterone in adult men.^20^, ^21^ It is also plausible that systemic inflammation and fever caused by infectious diseases may also impact Leydig cell development and function. Here, we have used childhood questionnaire data from the ALSPAC cohort to determine if common childhood infections might influence Leydig cell development, leading to long‐term impacts on the adult Leydig cell functional capacity, as represented by peripheral INSL3 concentration. For chickenpox (Table 3A), a marked association is found between early chickenpox infection and circulating INSL3 concentration at both 17 and 24 years. No relationship was found for measles (Table 3B), though incidence was low. Because of low incidence, only data reflecting total incidence before 157 months (13 years) are shown; earlier time‐points also failed to indicate any association (not shown). A positive association was observed for mumps and possibly for meningitis (though incidence was very low, ≤ 5 cases) with INSL3 at 17 years, though not at 24 years (Table 3B). Conversely, an association was noted for whooping cough and possibly also for scarlet fever, but only with INSL3 at 24 years (Table 3B). Taken together, these data imply that common childhood infections may individually lead to a reduction of circulating INSL3 concentration in young adulthood of between 10% and 17%.

**TABLE 3:** Influence of childhood infections on circulating INSL3 concentration at 17 and 24 years of age.

A. Chickenpox						
	INSL3 at 17 years			INSL3 at 24 years		
	Mean ± SD	*N*	*p*‐value	Mean ± SD	*N*	*p*‐value
Before 18 months						
yes	1.29 ± 1.00	239	**0.034**	1.99 ± 1.17	213	**0.017**
no	1.46 ± 1.50	932		2.22 ± 1.60	892	
Before 30 months						
yes	1.32 ± 1.04	370	**0.008**	1.99 ± 1.22	342	**0.002**
no	1.53 ± 1.70	773		2.28 ± 1.67	732	
Before 42 months						
yes	1.39 ± 1.09	608	0.054	2.13 ± 1.44	550	0.190
no	1.60 ± 2.39	545		2.26 ± 1.74	536	
54–69 months						
yes	1.43 ± 1.08	172	0.683	2.22 ± 1.37	178	0.658
no	1.47 ± 1.62	926		2.17 ± 1.56	847	
69–81 months						
yes	1.46 ± 1.08	48	0.968	2.25 ± 1.23	43	0.777
no	1.46 ± 1.57	1020		2.19 ± 1.57	960	
79–91 months						
yes	1.30 ± 0.99	31	0.253	2.29 ± 1.17	28	0.659
no	1.52 ± 1.91	1063		2.16 ± 1.54	1004	
91–103 months						
yes	1.13 ± 0.93	12	0.231	2.12 ± 0.80	13	0.721
no	1.47 + 1.57	1070		2.21 ± 1.61	991	
116–128 months						
yes	1.29 + 0.92	7	0.744	1.99 ± 0.91	6	0.752
no	1.48 + 1.60	1029		2.20 ± 1.58	970	
Before 157 months						
yes	1.49 ± 1.66	913	0.943	2.17 ± 1.61	857	0.452
no	1.48 ± 1.35	43		2.36 ± 1.96	42	

B. Other childhood infections						
BEFORE 157 months	INSL3 at 17 years			INSL3 at 24 years		
	Mean ± SD	*N*	*p*‐value	Mean ± SD	*N*	*P*‐value
Measles						
yes	1.58 ± 1.80	64	0.612	2.08 ± 2.09	56	0.677
no	1.48 ± 1.60	932		2.20 ± 1.55	912	
Mumps						
yes	1.12 ± 0.83	29	**0.028**	2.60 ± 2.71	32	0.403
no	1.49 ± 1.62	1006		2.19 ± 1.57	941	
Whooping cough						
yes	2.16 ± 3.26	17	0.398	1.81 ± 0.65	19	**0.023**
no	1.47 ± 1.57	1020		2.20 ± 1.62	955	
Meningitis*						
yes	0.80 ± 0.28	≤5*	**0.011** *	1.74 ± 0.66	≤5*	0.523*
no	1.49 ± 1.61	1032		2.20 ± 1.61	968	
Scarlet fever						
yes	1.58 ± 1.14	16	0.802	1.70 ± 0.90	14	0.059
no	1.48 ± 1.62	1017		2.20 ± 1.62	961	

*Notes*: (A) Chickenpox. (B) Other childhood infections. Categorical comparisons of questionnaire responses using *t*‐tests. Results highlighted in red are significant at *p* < 0.05.

* Because the *N* value is very small (≤ 5), this result and its *p*‐value are to be treated with caution.

### 3.4 Multiple regression analysis

Multiple regression analysis (stepwise backwards) was also carried out including all the above parameters which could be considered as non‐collinear and applying an inclusion threshold of *p* < 0.1 with exclusion at *p* < 0.05. Age at peak height velocity was also included. Except for INSL3 at 17 years, none were found to be significantly contributing to the overall variance of INSL3 concentration at 24 years.

## 4 DISCUSSION

Using correlation analysis, this study has assessed a wide range of maternal, neonatal, childhood, and adolescent parameters, including anthropometric, lifestyle, and biochemical ones, as well as incidence of childhood infectious disease, for their association with Leydig cell functional capacity in young adulthood represented by circulating INSL3. Of the many factors analysed, childhood BMI and early chickenpox infection stand out as potential determinants of adult Leydig cell functional capacity.

The steroidogenic Leydig cells of the testes are unusual in that once they are established in young adulthood this population of cells remains relatively stable without further cell division for the remainder of the male lifespan, and with only minimal cell attrition.^2^ Although androgen production itself is acutely very variable, the functional capacity of the Leydig cells to make these steroids remains consistent across the lifespan and can be assessed by measuring the circulating Leydig cell hormone INSL3. This constitutively secreted peptide varies little within an individual over months or years and effectively measures the number of Leydig cells and their average differentiation status.^4^ In large aging male cohorts, we have previously shown that despite very low within‐individual variance, there is high between‐individual variance and that the latter predicts age‐dependent morbidity and hypogonadism.^5^, ^7^, ^22^, ^23^ Whoever has low INSL3 in older age likely had reduced Leydig cell functional capacity and low INSL3 already as a younger man. However, we know very little about the factors influencing adult Leydig cell functional capacity, except that it seems to become established during puberty, when adult‐type Leydig cells proliferate and differentiate from testicular mesenchymal stem cells during childhood and adolescence.

The ALSPAC “Children of the Nineties” cohort is unique in offering for the first time the possibility of assessing those factors in early life which might be in part responsible for determining the final post‐pubertal Leydig cell functional capacity as represented by INSL3. We have first shown that at age 24 years INSL3 levels have stabilised to the young adult status, where acutely only BMI and smoking indicate small correlative effects,^10^ as noted previously for another young cohort.^6^ In contrast, at 17 years of age, puberty is still not complete with high INSL3 variance, both cross‐sectionally as well as longitudinally, and maximal INSL3 concentrations mostly not yet reached.^10^ Estimates suggest that, in this population, puberty attains maximal INSL3 expression at around 22 years, concomitant with a stabilisation of the Leydig cell population and adult‐type homeostatic regulation by the Hypothalamo‐Pituitary‐Gonadal (HPG) axis.

Here, we have applied bivariate correlation analysis to assess a range of potential influences from pregnancy, through infancy, childhood and adolescence, on both the late pubertal circulating INSL3 concentration at 17 years and that of young adulthood at 24 years. During pregnancy and infancy in the human, the adult Leydig cell population is represented only by a small pool of relatively undifferentiated stem cells within the testes.^1^, ^2^ Later, at around 8–9 years of age, these will begin to proliferate and differentiate under the influence of pituitary LH to mark the beginning of pubertal development in boys. Thus, any early (< 8 years) influences on Leydig cell development are likely to be indirect or on the stem cell population. As Table 1 indicates, only birthweight and gestational age suggest any influence (negatively) and then only on INSL3 at 17 years, but not on the INSL3 concentration at 24 years, though birthweight also correlates with later BMI (Table S1) so may not be causative in this context. These correlation coefficients are small, accounting for maximally 0.4% of the INSL3 variance, so may be considered as not clinically relevant. None of the possible factors known to influence the male phenotype show any effect. This includes the foetus being SGA, placental weight, maternal smoking, both before pregnancy and during the first trimester, estrogenic influences manifest as an altered 2D/4D ratio, and early infant feeding patterns.

Histologically, pubertal progression, beginning at around 8–9 years of age and ending at some time after 17 years, is manifest as increasing Leydig cell proliferation and differentiation over this period. Initially, peritubular mesenchymal stem cells become activated and change into so‐called Leydig progenitor cells, then immature Leydig cells, and finally adult‐type mature Leydig cells. These last are characterised by the production of INSL3. This process of proliferation and differentiation is induced and stimulated by pulses of pituitary LH, which increase in both amplitude and frequency until the end of puberty, when homeostatic mechanisms, caused chiefly by testosterone and its aromatisation to estradiol, act back on the pituitary and hypothalamus to regulate the pulse‐generator. This ultimately leads to a sub‐maximal steady‐state of the Leydig cell population^24^ which appears to represent a constant within any one individual for the remainder of the lifespan. Circulating INSL3 effectively measures this adult steady‐state. Extensive bivariate correlation analysis (Table 2) shows no evidence of a relationship until 9 to 10 years of age, when there is a first impact of BMI on circulating INSL3 at both 17 and 24 years. This negative association is then strongly manifest at 13 years, which represents more‐or‐less the age at peak height velocity for this population.^10^ A negative influence of childhood and adolescent overweight on later adult morbidity, such as kidney disease, diabetes, or some cancers, has been reported previously,^25^, ^26^ though the causative relationship here is not clear.

Interestingly, not only BMI, but also height itself at 13 years appears to relate to INSL3 at 17, here positively. Together, such findings suggest that growth rate per se, negatively influenced by increasing BMI, could be a factor in determining the final functional capacity of the Leydig cell population. This may explain also why free T4 (thyroxine), though not T3 (triiodothyronine) or TSH, at 15 years of age also indicates a positive association with INSL3 at 17 years, since at this age the thyroid activity is closely linked to skeletal growth.^27^ This may also relate to the positive association of INSL3 at 17 years (but not at 24 years) with spot cortisol levels at 8.5 and 15 years (Table 2E). These cortisol values likely reflect the age of adrenarche in these boys and may be co‐regulated with the HPG axis and hence Leydig cell development. That early/pre‐ puberty might represent a sensitive window for Leydig cell development has already been suggested by studies in bulls, where it was shown that only by enriching for protein in the early/pre‐pubertal food regimen, though not later, could the pubertal trajectory and INSL3 production be advanced.^28^

A further important finding from this study relates to the negative association of some childhood viral and bacterial infections with later Leydig cell functional capacity (Table 3). In particular, chickenpox acquired prior to 30 months of age is associated with a marked 10%–14% reduction in mean INSL3 concentration both at 17 and at 24 years of age (Table 3A). Childhood mumps or possibly meningitis infection led to a similar reduction in INSL3 concentration at 17, though not at 24 years. And childhood whooping cough as well as possibly scarlet fever, though rare, also led to a 17.5% reduction in INSL3 in the 24 year‐old young men (Table 3B). Since, although still in a very dynamic state, the INSL3 concentration at 17 years is still a major predictor of INSL3 concentration at 24 years,^10^ we can assume that lack of a statistical relationship at either 17 or 24 years with regard to childhood infection is probably due to the very small numbers of cases reported.

It is now well established that some viral infections in adult men may target the Leydig cells of the testis. Especially research on HIV and COVID‐19 in men and animal models has shown that these cells possess the necessary viral receptors to import the virus into the cells where they can disrupt the Leydig cell function, leading to contemporary reduction in testosterone production.^20^, ^21^ It is also recorded that mumps infection in young men may cause a secondary inflammation of the testes in the form of acute orchitis,^29^ with rare cases of chickenpox in adult men also suggesting that this virus can induce orchitis.^30^ This is the first study to show that particularly early childhood infections (both viral and bacterial) could permanently affect the Leydig cell population in the testes, either directly or consequent on inflammation and fever, potentially leading to a reduction in functional capacity and hence possibly to later hypogonadism. The timing of infection suggests that it is both the early progenitor and stem cell stages of Leydig cell differentiation, as well as early intermediates, which are being most affected. Taken together, these results strongly support the use of appropriate vaccination in young children now additionally to alleviate a possible risk of later reduced androgen production.

Longitudinal epidemiological studies such as this one are at best suited to proposing novel hypotheses and concepts, which can be evaluated in more targeted studies, clinical trials, or laboratory experiments. They can suggest relative risk factors but are not designed to elaborate causality. Multiple regression analysis using parameters which may individually correlate with INSL3, but which are not collinear, failed to indicate any significant, potentially causal contributors, implying that the associations identified in this study, whilst significant, may only be contributing to a minor degree to the adult variance in INSL3. Nevertheless, the application here of a parameter, adult INSL3 concentration, which is constitutive and shows high within‐individual consistency over years and into older age, means that correlations with earlier childhood and adolescent parameters, such as those collected for the ALSPAC cohort, offer a high level of plausibility as potentially causative factors. Adult testosterone production could not have offered such insight, since this hormone (and unlike INSL3) is highly variable within an individual, being continuously and acutely modulated by the homeostatic control of the HPG axis and the many factors affecting it.

This study has identified early adolescent BMI (ca. 11–13 years) as impacting adult Leydig cell functional capacity, as also relative growth around that time‐period in a boy's life. It has also shown that early childhood infection, for example, by chickenpox, whooping cough, mumps, or meningitis, may also be a negative determinant for Leydig cell functional capacity in young men. Maternal and related factors appear to have less influence, possibly because at that period in a child's life, adult‐type Leydig cells are represented only by a few testicular stem cells, the Leydig cells in the male fetus being of quite discrete embryological origin.^2^ This retrospective study has substantial limitations, in the numbers of boys assessed, as well as in the nature and range of parameters that were measured. Future research will need to validate the findings presented here and assess other parameters, including further endocrine, as well as genetic and epigenetic aspects.

## CONFLICT OF INTEREST STATEMENT

The authors declare that the research was conducted in the absence of any commercial or financial relationships that could be construed as a potential conflict of interest.

## Supporting information




**TABLE S1** Bivariate correlation analysis of overweight and obesity‐related parameters. See Section 2 for details of all parameters. Results highlighted in red are significant at *p* < 0.05.
