# Supplementary material for: Maternal, childhood and adolescent influences on Leydig cell functional capacity and circulating INSL3 concentration in young adults: Importance of childhood infections and body mass index
Source: Andrology. 2025 Jul 2;14(5):1172–82. doi: 10.1111/andr.70091 (PMC13266448; doi:10.1111/andr.70091)
Supplement: Supplementary file 1 — TABLE S1 Bivariate correlation analysis of overweight and obesity‐related parameters. See Section 2 for details of all parameters. Results highlighted in red are significant at p < 0.05. [file ANDR-14-1172-s001.docx]

|  | | **BMI 8y** | **BMI 9y** | **BMI 10y** | | **BMI 11y** | **BMI 13y** | **BMI 14y** | | **BMI 16y** | **BMI 17y** | **wt.gain**  **B – 6w** | **wt.gain**  **B - 9m** | **wt.gain**  **B - 18m** | **wt.gain**  **6w - 9m** | | **wt.gain**  **6w-18m** | **wt.gain**  **9m-18m** | **weaning**  **index** | **kcal int. 3y** | **kcal int. 7y** | **kcal int. 13y** |
| --- | --- | --- | --- | --- | --- | --- | --- | --- | --- | --- | --- | --- | --- | --- | --- | --- | --- | --- | --- | --- | --- | --- |
| **birthweight** | Pearson correlation | 0.057 | 0.179 | | 0.180 | 0.185 | 0.152 | 0.145 | 0.192 | | 0.155 | 0.052 | 0.009 | 0.000 | | 0.026 | 0.004 | 0.012 | 0.013 | 0.245 | 0.226 | 0.150 |
|  | Significance (p-value) | 0.100 | <0.001 | | <0.001 | <0.001 | <0.001 | <0.001 | <0.001 | | <0.001 | 0.030 | 0.703 | 0.998 | | 0.273 | 0.966 | 0.614 | 0.622 | <0.001 | <0.001 | <0.001 |
|  | N | 827 | 917 | | 901 | 914 | 932 | 934 | 894 | | 885 | 1759 | 1759 | 1759 | | 1759 | 1759 | 1759 | 1469 | 1732 | 1732 | 17632 |
| **BMI 8y** | Pearson correlation | 1 | 0.352 | | 0.242 | 0.316 | 0.266 | 0.282 | 0.262 | | 0.240 | 0.014 | 0.003 | 0.015 | | 0.011 | 0.020 | 0.019 | 0.001 | 0.170 | 0.182 | 0.159 |
|  | Significance (p-value) |  | <0.001 | | <0.001 | <0.001 | <0.001 | <0.001 | <0.001 | | <0.001 | 0.678 | 0.933 | 0.669 | | 0.756 | 0.567 | 0.584 | 0.980 | <0.001 | <0.001 | <0.001 |
|  | N |  | 601 | | 591 | 595 | 575 | 569 | 532 | | 511 | 834 | 834 | 834 | | 834 | 834 | 834 | 739 | 834 | 834 | 834 |
| **BMI 9y** | Pearson correlation |  | 1 | | 0.780 | 0.771 | 0.664 | 0.663 | 0.691 | | 0.621 | 0.025 | -0.016 | -0.023 | | 0.003 | -0.017 | -0.026 | -0.087 | 0.402 | 0.411 | 0.321 |
|  | Significance (p-value) |  |  | | <0.001 | <0.001 | <0.001 | <0.001 | <0.001 | | <0.001 | 0.444 | 0.636 | 0.485 | | 0.934 | 0.596 | 0.428 | 0.012 | <0.001 | <0.001 | <0.001 |
|  | N |  |  | | 645 | 642 | 624 | 605 | 572 | | 561 | 924 | 924 | 924 | | 924 | 924 | 924 | 831 | 924 | 924 | 924 |
| **BMI 10y** | Pearson correlation |  | | | 1 | 0.832 | 0.701 | 0.709 | 0.711 | | 0.664 | -0.020 | 0.001 | -0.077 | | -0.002 | -0.073 | -0.074 | -0.040 | 0.433 | 0.459 | 0.396 |
|  | Significance (p-value) |  |  |  |  | <0.001 | <0.001 | <0.001 | <0.001 | | <0.001 | 0.538 | 0.973 | 0.020 | | 0.952 | 0.027 | 0.027 | 0.258 | <0.001 | <0.001 | <0.001 |
|  | N |  |  |  |  | 658 | 642 | 619 | 580 | | 564 | 909 | 909 | 909 | | 909 | 909 | 909 | 801 | 909 | 909 | 909 |
| **BMI 11y** | Pearson correlation |  | | | | 1 | 0.684 | 0.751 | 0.701 | | 0.645 | 0.006 | -0.031 | -0.030 | | -0.007 | -0.024 | -0.032 | -0.036 | 0.430 | 0.453 | 0.378 |
|  | Significance (p-value) |  |  |  |  |  | <0.001 | <0.001 | <0.001 | | <0.001 | 0.855 | 0.346 | 0.364 | | 0.822 | 0.474 | 0.334 | 0.298 | <0.001 | <0.001 | <0.001 |
|  | N |  |  |  |  |  | 653 | 619 | 582 | | 570 | 922 | 922 | 922 | | 922 | 922 | 922 | 824 | 921 | 921 | 921 |
| **BMI 13y** | Pearson correlation |  | | | | | 1 | 0.714 | 0.640 | | 0.644 | 0.003 | 0.013 | -0.020 | | 0.034 | -0.012 | -0.005 | -0.032 | 0.365 | 0.376 | 0.325 |
|  | Significance (p-value) |  |  |  |  |  |  | <0.001 | <0.001 | | <0.001 | 0.922 | 0.699 | 0.549 | | 0.301 | 0.720 | 0.886 | 0.365 | <0.001 | <0.001 | <0.001 |
|  | N |  |  |  |  |  |  | 658 | 596 | | 578 | 938 | 938 | 938 | | 938 | 938 | 938 | 829 | 938 | 938 | 938 |
| **BMI 14y** | Pearson correlation |  | | | | | | 1 | 0.789 | | 0.743 | 0.024 | 0.012 | -0.030 | | 0.024 | -0.026 | -0.014 | -0.038 | 0.353 | 0.371 | 0.347 |
|  | Significance (p-value) |  |  |  |  |  |  |  | <0.001 | | <0.001 | 0.463 | 0.710 | 0.363 | | 0.454 | 0.429 | 0.668 | 0.275 | <0.001 | <0.001 | <0.001 |
|  | N |  |  |  |  |  |  |  | 669 | | 646 | 942 | 942 | 942 | | 942 | 942 | 942 | 819 | 942 | 942 | 942 |
| **BMI 16y** | Pearson correlation |  | | | | | | | 1 | | 0.830 | -0.005 | -0.014 | -0.045 | | -0.017 | -0.049 | -0.047 | -0.036 | 0.359 | 0.368 | 0.325 |
|  | Significance (p-value) |  |  |  |  |  |  |  |  | | <0.001 | 0.892 | 0.681 | 0.174 | | 0.604 | 0.141 | 0.154 | 0.311 | <0.001 | <0.001 | <0.001 |
|  | N |  |  |  |  |  |  |  |  | | 664 | 905 | 905 | 905 | | 905 | 905 | 905 | 783 | 904 | 904 | 904 |
| **BMI 17y** | Pearson correlation |  | | | | | | | | | 1 | 0.012 | 0.040 | -0.046 | | 0.033 | -0.050 | -0.033 | 0.011 | 0.374 | 0.388 | 0.363 |
|  | Significance (p-value) |  |  |  |  |  |  |  |  |  |  | 0.712 | 0.234 | 0.168 | | 0.324 | 0.136 | 0.324 | 0.764 | <0.001 | <0.001 | <0.001 |
|  | N |  |  |  |  |  |  |  |  |  |  | 896 | 896 | 896 | | 896 | 896 | 896 | 781 | 895 | 895 | 895 |
| **wt.gain**  **B – 6w** | Pearson correlation |  | | | | | | | | | | 1 | 0.355 | 0.169 | | 0.689 | 0.335 | 0.182 | 0.034 | 0.005 | 0.007 | 0.015 |
|  | Significance (p-value) |  |  |  |  |  |  |  |  |  |  |  | <0.001 | <0.001 | | <0.001 | <0.001 | <0.001 | 0.192 | 0.838 | 0.783 | 0.523 |
|  | N |  |  |  |  |  |  |  |  |  |  |  | 1781 | 1781 | | 1781 | 1781 | 1781 | 1488 | 1754 | 1754 | 1754 |
| **wt.gain**  **B - 9m** | Pearson correlation |  | | | | | | | | | | | 1 | 0.207 | | 0.850 | 0.218 | 0.406 | 0.022 | 0.027 | 0.033 | 0.045 |
|  | Significance (p-value) |  |  |  |  |  |  |  |  |  |  |  |  | <0.001 | | <0.001 | <0.001 | <0.001 | 0.400 | 0.265 | 0.166 | 0.057 |
|  | N |  |  |  |  |  |  |  |  |  |  |  |  | 1781 | | 1781 | 1781 | 1781 | 1488 | 1754 | 1754 | 1754 |
| **wt.gain**  **B - 18m** | Pearson correlation |  | | | | | | | | | | | | 1 | | 0.205 | 0.964 | 0.947 | 0.032 | -0.001 | -0.003 | 0.017 |
|  | Significance (p-value) |  |  |  |  |  |  |  |  |  |  |  |  |  | | <0.001 | <0.001 | <0.001 | 0.211 | 0.978 | 0.916 | 0.480 |
|  | N |  |  |  |  |  |  |  |  |  |  |  |  |  | | 1781 | 1781 | 1781 | 1488 | 1754 | 1754 | 1754 |
| **wt.gain**  **6w - 9m** | Pearson correlation |  | | | | | | | | | | | | | | 1 | 0.319 | 0.373 | 0.027 | 0.030 | 0.039 | 0.052 |
|  | Significance (p-value) |  |  |  |  |  |  |  |  |  |  |  |  |  |  |  | <0.001 | <0.001 | 0.301 | 0.205 | 0.105 | 0.030 |
|  | N |  |  |  |  |  |  |  |  |  |  |  |  |  |  |  | 1781 | 1781 | 1488 | 1754 | 1754 | 1754 |
| **wt.gain**  **6w-18m** | Pearson correlation |  | | | | | | | | | | | | | | | 1 | 0.922 | 0.039 | -0.003 | -0.003 | 0.015 |
|  | Significance (p-value) |  |  |  |  |  |  |  |  |  |  |  |  |  |  |  |  | <0.001 | 0.135 | 0.894 | 0.886 | 0.531 |
|  | N |  |  |  |  |  |  |  |  |  |  |  |  |  |  |  |  | 1781 | 1488 | 1754 | 1754 | 1754 |
| **wt.gain**  **9m-18m** | Pearson correlation |  | | | | | | | | | | | | | | | | 1 | 0.035 | 0.000 | 0.000 | 0.026 |
|  | Significance (p-value) |  |  |  |  |  |  |  |  |  |  |  |  |  |  |  |  |  | 0.183 | 0.989 | 0.994 | 0.268 |
|  | N |  |  |  |  |  |  |  |  |  |  |  |  |  |  |  |  |  | 1488 | 1754 | 1754 | 1754 |
| **weaning**  **index** | Pearson correlation |  | | | | | | | | | | | | | | | | | 1 | -0.031 | -0.028 | -0.032 |
|  | Significance (p-value) |  |  |  |  |  |  |  |  |  |  |  |  |  |  |  |  |  |  | 0.233 | 0.282 | 0.215 |
|  | N |  |  |  |  |  |  |  |  |  |  |  |  |  |  |  |  |  |  | 1480 | 1480 | 1480 |
| **kcal int. 3y** | Pearson correlation |  | | | | | | | | | | | | | | | | | | 1 | 0.967 | 0.739 |
|  | Significance (p-value) |  |  |  |  |  |  |  |  |  |  |  |  |  |  |  |  |  |  |  | <0.001 | <0.001 |
|  | N |  |  |  |  |  |  |  |  |  |  |  |  |  |  |  |  |  |  |  | 1754 | 1754 |
| **kcal int. 7y** | Pearson correlation |  | | | | | | | | | | | | | | | | | | | 1 | 0.810 |
|  | Significance (p-value) |  |  |  |  |  |  |  |  |  |  |  |  |  |  |  |  |  |  |  |  | <0.001 |
|  | N |  |  |  |  |  |  |  |  |  |  |  |  |  |  |  |  |  |  |  |  | 1754 |

**Supplement Table 1 – Bivariate correlation analysis of overweight and obesity related parameters**

**Supplement Table 1**

Bivariate correlation analysis of overweight and obesity-related parameters. See Methods and Materials for details of all parameters. Results highlighted in red are significant at p<0.05.
